# Seasonal variations on prevalence and antimicrobial resistance of Salmonella isolated from dogs in Khon Kaen province, Northeast Thailand

**DOI:** 10.1186/2047-2994-4-S1-P140

**Published:** 2015-06-16

**Authors:** P Sringam, S Angkititrakul

**Affiliations:** 1 Physiology, Khon Kaen University, Khon Kaen, Thailand; 2 Veterinary Public Health, Khon Kaen University, Khon Kaen, Thailand

## Introduction

S*almonella spp.* can be isolated from healthy dogs at rates of up to 36% and they tend to shed *Salmonella* organisms to feces for very prolonged periods of time after infection.

## Objectives

To describe *Salmonella* isolated from dogs and to investigate their antimicrobial resistance based on seasons in Khon Kaen province, Thailand.

## Methods

During 2012-2013, 428 fecal samples were collected from dogs by rectal swab in 3 seasons (winter, summer and rainy). All samples were examined for *Salmonella* spp. isolation and identification by ISO 6597:2002. To assess the prevalence of antimicrobial resistant patterns was done using disk diffusion technique among 7 antimicrobials.

## Results

*Salmonella* contaminated to dogs feces in winter, summer and rainy seasons were 13.6%, 13.1% and 9.3%, respectively. The identified most found serovars in winter, summer and rainy seasons were *S*. Give (23.5%), *S*. Typhimurium (40%) and *S*. Weltevreden (21.4%), respectively. Highly resistant amplicillin, sulfamethoxazole/trimetroprim and tetracycline *Salmonella* spp. isolated from dogs were 52%, 24% and 48%, respectively.

## Conclusion

*Salmonella* spp. can be detected in dogs without any overt clinical signs indicating possible carrier state that can be spread to their owners especially in children. Therefore, to avoid carrier state in pets, sanitary and health management is crucial for their owners. Ongoing multi-provincial investigation should be encouraged to better understand the reasons for these observed seasonal variations.

## Disclosure of interest

None declared.
